# Effectiveness of postoperative oral administration of cephalexin and metronidazole on surgical site infection among obese women undergoing cesarean section: a randomized, double-blind, placebo-controlled, parallel-group study—phase III

**DOI:** 10.1186/s13756-022-01191-y

**Published:** 2022-12-05

**Authors:** Fatemeh Tara, Sina Danesteh, Maral Rezaee, Kiarash Roustai Geraylow, AmirAli Moodi Ghalibaf, Somayeh Moeindarbari

**Affiliations:** 1 Faculty of Medicine, Mashhad University of Medical Sciences, Mashhad, Iran; 2 Student Research Committee, Faculty of Medicine, Mashhad University of Medical Sciences, Mashhad, Iran; 3 Student Research Committee, Semnan University of Medical Sciences, Semnan, Iran; 4 Student Research Committee, Faculty of Medicine, Birjand University of Medical Sciences, Birjand, Iran; 5 Department of Obstetrics and Gynecology, Faculty of Medicine, Mashhad University of Medical Sciences, Mashhad, Iran

**Keywords:** Cephalexin, Metronidazole, Surgical site infection, Cesarean section, Obesity

## Abstract

**Background:**

Cesarean section (CS) is the most frequently performed surgery in the United States. Compared to vaginal delivery, CS has a higher risk of maternal and neonatal mortality, morbidities, and complications, among which surgical site infection (SSI) is the most common. We aimed at evaluating the effectiveness of postoperative oral administration of cephalexin and metronidazole on SSI among obese women undergoing CS.

**Methods:**

We conducted a randomized, double-blind clinical trial comparing the prophylactic effect of oral cephalexin and metronidazole vs cephalexin and placebo on SSI following CS among obese women. who had received preoperative prophylactic cephalosporin antibiotics. The study was conducted at the Ommolbanin Hospital, affiliated with Mashhad University of Medical Sciences from April 2019 to February 2020.

**Result:**

The participants were randomized into the intervention group (n = 210) and the control group (n = 210). At week-1 follow-up, the outcomes were significantly lower in the intervention group as compared to the control group in terms of fever (9% vs 19%, *p* = 0.003), abnormal discharge from the incision (serous: 8.6% vs 10.5%, purulent: 2.9% vs 16.7%, *p* < 0.001), incision separation (1% vs 7.1%, *p* = 0.001), and cellulitis (4.8% vs 13.3%, *p* = 0.002). At week-2 follow-up, there were no patients in the intervention group with fever, abnormal discharge from the incision, incision separation, or cellulitis and there was a statistically significant difference for fever, abnormal discharge from the incision, and incision separation between the two groups (*p* < 0.001, *p* = 0.001, *p* = 0.014, respectively).

**Conclusion:**

Post-operative administration of cephalexin and metronidazole for 48-h post-cesarean delivery among obese women, in addition to the standard pre-operative prophylaxis, reduced the overall rate of surgical site infection and wound infection symptoms in a 2-week follow-up.

*Trial registration* The study protocol was approved by the Iranian Registry of Clinical Trials (IRCTID: IRCT20200608047685N2) on 2021-03-15.

## Bcakground

Cesarean section (CS) is the most frequently performed surgery in the United States, accounting for 1.2 million cases annually, which constitutes 31.7% of all births [1]. World Health Organization (WHO) recommends that the rate of CS should not exceed 10–15% of total deliveries, while this proportion is reported to be as high as 48% in Iran [2, 3]. Despite health policies on increasing public awareness, the frequency of CS has dramatically increased during the past decades without any medical indication and has become a severe concern for health systems in many countries. This concern is warranted because compared to vaginal delivery, CS has a higher risk of maternal and neonatal mortality, morbidities, and complications, among which surgical site infection (SSI) is a common complication with an approximate frequency of 10% [4].

There are several predisposing factors for SSI following CS such as duration of operation (the longer the duration, the higher the risk of SSI), hypertensive disorders, emergency CS, diabetes, pre-existing infection, chorioamnionitis, and elevated intraoperative blood loss; and the most significant independent risk factors reported by previous studies are being overweight (body mass index [BMI] 25–30 kg/m(2)) and obese (BMI 30–35 kg/m(2)) [4–8].

In addition to the clinical complications associated with SSI following CS, such as maternal and neonatal morbidity and mortality, these infections lead to significant increases in the duration of hospitalization and health care costs; estimated to be as high as $3,700 per case [9, 10].

Routine use of preoperative prophylactic cephalosporin antibiotics has been reported to decrease the occurrence of SSI following CS, but few studies have specifically addressed optimal antibiotic regimens in the obese population [11–13].

The objective of our study was to evaluate the prophylactic effect of postoperative oral administration of cephalexin and metronidazole on surgical site infection among obese women undergoing CS.

## Methods

After the approval of the study protocols by the Mashhad University of Medical Sciences Institutional Review Board, we conducted a single-center, double-blind, randomized clinical trial to determine the effectiveness of postoperative oral administration of cephalexin plus metronidazole compared with placebo for 48 h after CS for the prevention of SSI among obese women. Participants were recruited from April 2019 to February 2020 at the Ommolbanin Hospital, affiliated with Mashhad University of Medical Sciences. Written informed consent was obtained from all patients postoperatively.

Women were eligible for randomization if they aged at least 15 years, lived in Mashhad, had a pregnancy BMI of 30 or higher, had a final plan for cesarean delivery at Ommolbanin Hospital, and were able to come to the Ommolbanin Hospital for follow-up. Besides, both elective and emergency CS were recruited in the study. Patients were excluded if they had to take antibiotics in the postpartum period for any reason or had a preterm delivery, multiple births, amniorrhexis, immunodeficiency syndromes, known or suspected allergies to cephalexin or metronidazole, or diabetes mellitus needing insulin therapy.

All subjects gave written informed consent to participate in the study and then were randomized to the intervention group, receiving both 500 mg oral cephalexin and 500 mg oral metronidazole every 8 h for 6 doses; and the placebo group.

The CS was performed in standard practice by residents, fellows, and attending physicians. Prior to the skin incision, the abdomen was cleansed using the povidone-iodine solution, standard sterile draping was performed and prophylactic Cefazolin (2 g) was injected intravenously. Pfannenstiel skin incision was performed for all cases and uterine was incised using Kerr incision. Surgical dressings were removed 24 h postoperatively and the incision was irrigated using a normal saline solution. The surgical site was cleansed using povidone-iodine solution 48 h postoperatively.

Participants were randomly assigned to intervention and control groups using the simple randomization procedure. Inside 210 opaque, sealed and stapled envelopes was the word T (treatment group) and inside another 210 opaque, sealed and stapled envelopes was the word P (placebo group). All subjects were asked to pick an envelope and the card inside told if the patient was to be in the treatment or placebo group. Only the statistical analyzer was known about what each envelope included.

Cephalexin and metronidazole and their indistinguishable placebos were put in packs named T (treatment group) and P (placebo group) according to a computer-generated randomization list. In terms of appearance and shape, the placebo was exactly the same as the original antibiotics, in the way that placebo metronidazole was exactly the same as metronidazole in the form of a white round tablet and placebo cephalexin was exactly the same as the original antibiotic, as a yellow capsule.

First doses of oral Cephalexin, 500 mg, and oral Metronidazole, 500 mg, were administered 8 h after preoperative intravenous injection of prophylactic Cefazolin (2 g) and were continued every 8 h for another 5 doses.

Cephalexin, Metronidazole, Cephalexin placebo and Metronidazole placebo were all indistinguishable and produced by the Sobhan Pharmaceutical Company®.

Follow-up examinations at one week and two weeks postpartum were performed by the gynecologist of the study. All subjects were examined for drug adverse effects and symptoms of SSI such as fever (temperature equal to or greater than 37.5 Celsius), cellulitis, uterine tenderness, and wound separation.

Independent t-test or Mann– Whitney test was used to examine the differences of normally-distributed quantitative variables between the two groups. The Chi-square test or Fisher's exact test was used to compare differences in qualitative variables between the two groups. *p*-values of less than 0.05 were considered to be statically significant. All statistical analyses were performed using the Statistical Package for Social Sciences (SPSS) version 23 (SPSS Inc., Chicago, IL, USA).

We estimated the sample size for our study assuming a baseline rate of SSI of 6.4% in the treatment group and 15.4% in the placebo group on the basis of a study conducted by Amy M. Valent et al. in 2017 [13]. We predicted a 50% lower risk of SSI in the treatment group than in the placebo group. To have 80% power to detect a 50% difference in the rates of SSI, we estimated that the study needed 188 participants in each group (α = 0.05). In order to accommodate a 10% rate of postoperative loss to follow-up, we anticipated enrolling 210 participants in each group.

The ethical approval for conducting this study was obtained from the ‘Ethical Committee of the Mashhad University of Medical Sciences (Registration code: IR.MUMS.MEDICAL.REC.1397.733). Also, the study protocol was approved by the Iranian Registry of Clinical Trials (IRCTID: IRCT20200608047685N2) on 2021-03-15.

## Results

**Fig. 1:**
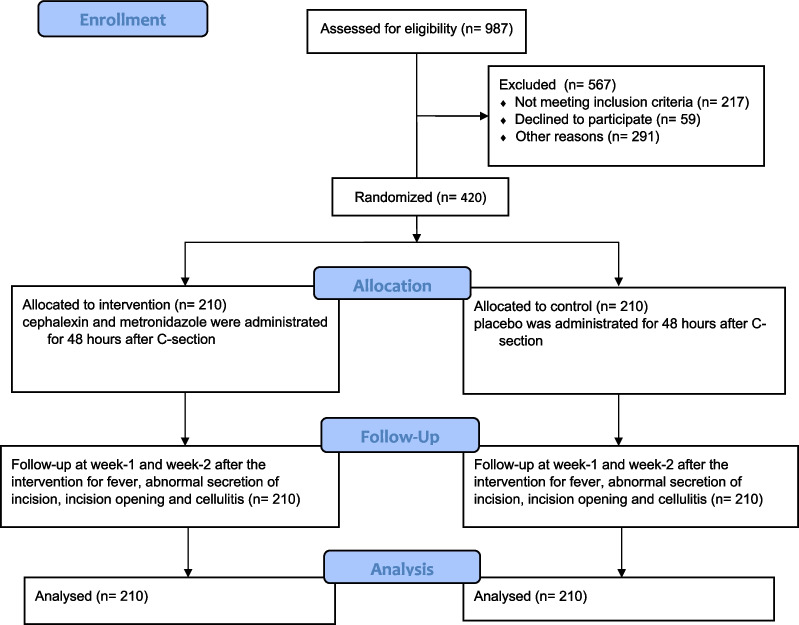
Flow diagram of the study

Among 987 participants, 567 were excluded due to not meeting inclusion criteria (n = 217), declined to participate (n = 59), and other reasons (n = 291), including either the cesarean section being performed in a non-standard way, forgetting the treatment staff to include the patients in the research plan, or the placebo was not available for a period of time. Four hundred and twenty participants were randomized into the intervention group (n = 210) and the control group (n = 210). Finally, all 420 participants were followed up and analyzed (Fig. 1).

The demographic data of the study participants are indicated in Table 1. The two groups were homogeneous in terms of the mean number of pregnancies, the number of cesarean sections, duration of cesarean section, body mass index, past surgical history, underlying diseases, medications, and C-section indications at the beginning of the study (*p* > 0.05).

**Table 1:** Participants characteristics

Variables	Intervention (n = 210)	Control (n = 210)	*p*-value
	N (%)	N (%)	
Pregnancy number (median)	4	4	0.920*
Past cesarean (median)	1	2	0.477*
Gestational age (weeks)	39	38	0.993*
Duration of C-section (min)	47.3	45.9	0.561*
BMI (kg/m^2^)	33.2	32.8	0.083*
Past surgery history			
Cesarean	146 (69.5)	157 (74.8)	0.166**
Appendectomy	7 (3.3)	4 (1.9)	0.781**
Cholecystectomy	3 (1.4)	4 (1.9)	0.210**
Laparoscopy	11 (5.2)	9 (4.3)	0.410**
Ovarian cystectomy	6 (2.9)	6 (2.9)	0.089**
Curettage	7 (3.3)	2 (1.0)	0.311**
Colporrhaphy	2 (1.0)	3 (1.4)	0.095**
No surgical history	28 (13.4)	25 (11.9)	0.510**
Underlying disease			
Gestational diabetes	15 (7.1)	19 (9.0)	0.177**
Epilepsy	4 (1.9)	5 (2.4)	0.981**
Gestational hypertension	9 (4.3)	8 (3.8)	0.870**
Thyroid disorders	11 (5.2)	16 (7.6)	0.561**
Asthma	5 (2.4)	10 (4.8)	0.210**
Anemia	7 (3.3)	7 (3.3)	0.957**
Pre-eclampsia	4 (1.9)	6 (2.9)	0.751**
No underlying disease	155 (73.8)	139 (66.2)	0.321**
Medications			
Enoxaparin	8 (3.8)	7 (3.3)	0.138**
Methyldopa	6 (2.9)	10 (4.8)	0.510**
Levothyroxine	11 (5.2)	16 (7.6)	0.091**
Ferrous sulfate	7 (3.3)	7 (3.3)	0.581**
Metformin	4 (1.9)	4 (1.9)	0.134**
Other	67 (31.9)	58 (27.6)	0.0881**
No drug	109 (51.9)	103 (49.0)	0.671**
Cesarean section indication			
Past C-section	114 (54.3)	107 (51.0)	0.311**
Fetal distress	17 (8.1)	17 (8.1)	0.410**
Multiple pregnancies	12 (6.2)	9 (4.3)	0.870**
Elective	2 (1.0)	4 (1.9)	0.651**
Prolonged first stage of labour	12 (6.2)	10 (5.2)	0.810**
Prolonged second stage of labour	5 (2.4)	5 92.4)	0.079**
Not responding to induction	11 (5.2)	11 (5.2)	0.095**
Breech presentation	6 (2.9)	13 (6.2)	0.310**
Pre-eclampsia	4 (1.9)	4 (1.9)	0.417**
CPD	5 (2.4)	3 (1.4)	0.639**
Macrosomia	9 (4.3)	6 (2.9)	0.061**
Hydrocephalic	5 (2.4)	3 (3.4)	0.123**
IUGR	4 (1.9)	3 (1.4)	0.077**
Placenta Previa	4 (1.9)	4 (1.9)	0.271**

*CPD* Cephalopelvic disproportion, *IUGR* Intrauterine growth restriction, *C-section* cesarean section, *BMI* Body mass index

*Mann–Whitney test/**Fisher's exact test

At week-1 follow-up, the outcomes were significantly lower in the intervention group as compared to the control group in terms of fever (9% vs 19%, *p* = 0.003), abnormal discharge from the incision (serous: 8.6% vs 10.5%, purulent: 2.9% vs 16.7%, *p* < 0.001), incision separation (1% vs 7.1%, *p* = 0.001), and cellulitis (4.8% vs 13.3%, *p* = 0.002), respectively (Table 2).

**Table 2:** Study Outcomes

Variables	Week 1		Week 2	
	Intervention (n = 210)	Control (n = 210)	Intervention (n = 210)	Control (n = 210)
	N (%)	N (%)	N (%)	N (%)
Fever (≥ 37.5 °C)	(9)19	(19)40	0 (0)	17 (8.1)
*p*-value	0.003*		< 0.001*	
Incision abnormal discharge				
Serous	18 (8.6)	22 (10.5)	0 (0)	10 (4.8)
Purulent	6 (2.9)	35 (16.7)	0 (0)	0 (0)
* p*-value	< 0.001**		0.001*	
Incision separation	2 (1)	15 (7.1)	0 (0)	6 (2.9)
* p*-value	0.001*		0.014*	
Cellulitis	10 (4.8)	28 (13.3)	0 (0)	2 (1)
* p*-value	0.002*		0.156*	

*Fisher's exact test/**Chi-square test

At the week 2 follow-up, there were no patients in the intervention group with fever, abnormal discharge from the incision, incision separation, or cellulitis. However, in the control group, fever, serous discharge, purulent discharge, incision separation, and cellulitis occurred in 8.1%, 4.8%, 0%, 2.9%, and 1.0% of the patients, respectively. There was a statistically significant difference in terms of fever, abnormal discharge, and incision separation between the two groups (*p* < 0.001, *p* = 0.001, *p* = 0.014, respectively). There were no significant differences between the two groups for cellulitis (*p* > 0.05) (Table 2).

## Discussion

In this randomized clinical trial among 420 obese women, we investigated the effect of post-cesarean section administration of oral cephalexin (500 mg) and metronidazole (500 mg) for 48 h in addition to the standard pre-operative antimicrobial prophylaxis (2 g of intravenous cefazolin before skin incision). Our results showed that postoperative administration of cephalexin and metronidazole significantly reduced wound infection symptoms, including fever, abnormal discharge from the incision, incision opening, and cellulitis within a week after delivery. By the second week, the results were more prominent. Among the participants in the treatment group, none of the study parameters were observed by week 2. The differences regarding fever, discharge, and incision separation were statistically significant between the two groups.

Surgical site infection (SSI) after cesarean delivery is a well-known complication and is estimated to occur in 1–10% of cesarean deliveries [14–16]. Prophylactic antibiotics are recommended for all women undergoing cesarean delivery in order to prevent infection [17]. Recommended antibiotics include the first generation cephalosporin (cefazolin) and, in patients with a beta-lactam allergy, the combination of clindamycin and an aminoglycoside [18]. Recently, the addition of wide-spectrum antibiotics such as azithromycin, gentamicin, and metronidazole to the routine use of cefazolin has gained a notable amount of attention [19].

Obesity is a known risk factor for the development of postcesarean delivery SSI [20]. It has been shown that physiological changes in obese patients reduce antibiotics blood concentration and penetration at the surgical site due to decreased tissue vascularity [15]. In addition, an increase in BMI has been associated with a decrease in cefazolin concentration in adipose tissues [21]. Therefore, obese patients are more likely to receive a subtherapeutic dosage of antibiotics [15]. Cephalexin and metronidazole have a wide-spectrum coverage and a high oral bioavailability and are well tolerated, making the combination as a good candidate for post-delivery prophylaxis [13].

Similar to our findings, a randomized control trial conducted on 403 obese women undergoing cesarean delivery showed a 48-h post-operative administration of oral cephalexin-metronidazole in addition to the standard treatment (pre-operative cefazolin), significantly reduced the rate of infection and cellulitis in the 30-day follow-up compared to the placebo group. However, they reported no significant decrease in fever, incisional morbidity, incision separation, and endometritis [13]. The participants for the previously mentioned study were enrolled in a 5-year period. Therefore, due to the differences in surgical techniques and SSI prevention methods used at the start of the study and those used in today's practice, their results could be altered [14]. In our study, participants were enrolled in a 10-month period. In addition, our study participants were evaluated at week-1 and week-2 of post-delivery, whereas in the previously mentioned study, patients were followed up once at day-30 post-delivery. Talbot et al. showed that by stratifying patients undergoing cesarean delivery into high-risk and low-risk groups, in which BMI > 30 kg/m^2^ was considered as a major risk factor, and managing them according to their risk group (for the low-risk group, a prophylactic dose of pre-operative intravenous antibiotics was assessed, and in the high-risk group, prophylactic antibiotic agents were administrated pre-operatively and continued for 24 h post-operatively) reduced overall SSI rate [22]. Another randomized trial on 160 participants reported lower post-operative infections, duration of hospitalization, and medication cost by using perioperative metronidazole and cefazolin compared to cefazolin alone [23].

Our study had some limitations. Firstly, the single-center nature of our study and only enrolling obese patients have limited the generalizability of our results. Secondly, we did not assess subgroup comparisons. Moreover, we did not consider antibiotic side effects, detail C-section complications that may have affected the results, effects on the babies who were breastfed were not examined, and despite today, there are some recommendations about using 3 g of Cefazolin, at the time we designed the interventions the 24th edition of the Williams Obstetrics recommended 2 g of Cefazolin [24]. Therefore, further studies are required to investigate the possible role of cesarean delivery indications, prior cesarean delivery, and other factors that might affect the efficacy of the protocol described in our study in order to consider them in the decision to whether use this post-operative antimicrobial prophylaxis or not. The large sample size and the methodology (double-blinded RCT) of this study can be considered as its strong point.

## Conclusion

In conclusion, postoperative administration of cephalexin and metronidazole for 48-h post-cesarean delivery among obese women, in addition to the standard pre-operative prophylaxis, reduced the overall rate of surgical site infection and other factors, including fever, abnormal discharge from the incision, incision opening, and cellulitis within a 2-week follow-up. Further studies are required to investigate the efficacy of this combination and the factors that need to be considered in the decision to assess this post-operative antimicrobial prophylaxis protocol.

## Data Availability

The data that support the findings of this study are available on request from the corresponding author. The data are not publicly available due to privacy or ethical restrictions.

## Abbreviations

CS: Cesarean section
SSI: Surgical site infection
WHO: World Health Organization
BMI: Body mass index
SPSS: Statistical package for social sciences
RCT: Randomized control trial
